# Comprehensive Management With the ABC (Atrial Fibrillation Better Care) Pathway in Clinically Complex Patients With Atrial Fibrillation: A Post Hoc Ancillary Analysis From the AFFIRM Trial

**DOI:** 10.1161/JAHA.119.014932

**Published:** 2020-05-06

**Authors:** Marco Proietti, Giulio Francesco Romiti, Brian Olshansky, Deirdre A. Lane, Gregory Y. H. Lip

**Affiliations:** ^1^ Liverpool Centre for Cardiovascular Science University of Liverpool and Liverpool Heart & Chest Hospital Liverpool United Kingdom; ^2^ Department of Clinical Sciences and Community Health University of Milan Italy; ^3^ Geriatric Unit Fondazione IRCCS Ca’ Granda Ospedale Maggiore Policlinico Milan Italy; ^4^ Department of Internal Medicine and Medical Specialties Sapienza‐University of Rome Italy; ^5^ Division of Cardiovascular Medicine University of Iowa Hospitals and Clinics Iowa City IA; ^6^ Aalborg Thrombosis Research Unit Department of Clinical Medicine Aalborg University Aalborg Denmark

**Keywords:** atrial fibrillation, integrated care, outcomes research, Atrial Fibrillation, Quality and Outcomes

## Abstract

**Background:**

For patients with atrial fibrillation, a comprehensive care approach based on the Atrial fibrillation Better Care (ABC) pathway can reduce the occurrence of adverse outcomes. The aim of this paper was to investigate if an approach based on the ABC pathway is associated with a reduced risk of adverse events in “clinically complex” atrial fibrillation patients, including those with multiple comorbidities, polypharmacy, and prior hospitalizations.

**Methods and Results:**

We performed a post hoc analysis of the AFFIRM (Atrial Fibrillation Follow‐up Investigation of Rhythm Management) trial. The principal outcome was the composite of all‐cause hospitalization and all‐cause death. An integrated care approach (ABC group) was used in 3.8% of the multimorbidity group, 4.0% of the polypharmacy group, and 4.8%, of the hospitalized groups. In all “clinically complex” groups, the cumulative risk of the composite outcome was significantly lower in patients managed consistent with the ABC pathway versus non‐ABC pathway‐adherent (all *P*<0.05). Cox regression analysis showed a reduction of composite outcomes in ABC pathway‐adherent versus non‐ABC pathway‐adherent for multimorbidity (hazard ratio [HR], 0.61, 95% CI, 0.44–0.85), polypharmacy (HR, 0.68, 95% CI, 0.47–1.00), and hospitalization (HR, 0.59, 95% CI, 0.42–0.85) groups. Secondary analyses showed that the higher number of ABC criteria fulfilled the larger associated reduction in relative risk, even for secondary outcomes considered.

**Conclusions:**

Use of an ABC consistent pathway is associated with fewer major adverse events in patients with atrial fibrillation who have multiple comorbidities, use of polypharmacy, and prior hospitalization.

**Nonstandard Abbreviations and Acronyms**
ABC: Atrial fibrillation Better Care
AFFIRM: Atrial Fibrillation Follow‐up Investigation of Rhythm Management

See Editorial by Yao et al.

Patients affected with atrial fibrillation (AF) are at high risk for cardiovascular and noncardiovascular death.^1^, ^2^, ^3^, ^4^, ^5^, ^6^ This risk is associated with multiple comorbidities,^7^, ^8^ polypharmacy^6^, ^9^, ^10^, and prior hospitalization,^11^, ^12^ all features that characterize the “clinically complex” patient.

Evidence has emerged indicating that an integrated or holistic management approach in patients with AF can reduce mortality associated with AF.^13^ These data, together with the need to reduce major adverse events in AF patients beyond just the risk of ischemic stroke, have inspired new thinking regarding a multifaceted approach to AF management.^14^, ^15^, ^16^ The Atrial fibrillation Better Care (ABC) pathway has been proposed to streamline implementation of an integrated approach to managing patients with AF.^16^

The ABC pathway has 3 main pillars: **“A” A**void stroke (with **A**nticoagulants); **“B” B**etter symptom management, with patient‐centered decisions on rate or rhythm control; **“C” C**ardiovascular and **C**omorbidity risk optimization.^16^ Thus far, retrospective analyses have shown that management resembling the ABC pathway has been associated with reduction in AF‐related clinical outcomes.^17^, ^18^, ^19^ In a post hoc analysis derived from the AFFIRM (Atrial Fibrillation Follow‐up Investigation of Rhythm Management) trial, we previously showed how a clinical approach based on ABC pathway was associated with reduction in risk for major bleeding, all‐cause death, cardiovascular death, first hospitalization, first cardiovascular hospitalization, and multiple hospitalizations.^17^

Here, we investigate if an approach based on the ABC pathway is associated with reduced risk of adverse events in 3 prespecified subgroups of “clinically complex” AF patients at high risk for all‐cause death and other adverse outcomes, including those with multiple comorbidities, those taking multiple drugs (polypharmacy), and those hospitalized when AF was diagnosed.

## Methods

The authors declare that all supporting data and methods used to derive the results and the related findings are available within the article.

We considered patients enrolled in the AFFIRM trial.^20^, ^21^ The AFFIRM trial was approved by the University of Missouri Institutional Review Board (IRB); the database was obtained from the National Institutes of Health. The IRB for every participating center approved the study protocol and all patients entered the study after providing written informed consent. The study was performed according to the European Union Note for Guidance on Good Clinical Practice CPMP/ECH/135/95 and the Declaration of Helsinki.

Implementation of the ABC pathway in the AFFIRM trial cohort has been described in detail elsewhere.^17^ In brief, the “A” criterion was fulfilled if the patient had a time in therapeutic range ≥70%; the “B” criterion was fulfilled if the patient presented with 2 or fewer symptoms; the “C” criterion was fulfilled if the patient were properly managed for the concomitant cardiovascular comorbidities (hypertension, coronary artery disease, peripheral artery disease, previous stroke/transient ischemic attack, heart failure).^17^

We defined 3 groups of “clinically complex” patients, deemed at high risk for adverse AF‐related outcomes: (1) multimorbidity group: if the patient had 2 or more concomitant conditions,^22^ among the 11 listed in the AFFIRM case report form (myocardial infarction, heart failure, hypertension, cardiomyopathy, valvular heart disease, congenital heart disease, previous stroke/transient ischemic attack, peripheral arterial disease, diabetes mellitus, hepatic/renal disease, pulmonary disease); (2) polypharmacy group: if the patient used 5 or more drugs, as reported in a previous paper from the same cohort^6^; (3) hospitalization group: if the patient was hospitalized at the time of the index AF event, as was originally included in the AFFIRM study.

### Study Outcomes

The primary outcome considered was a composite of all‐cause hospitalization and all‐cause death. We separately considered all‐cause hospitalization and all‐cause death as independent outcomes. We also considered occurrence of cardiovascular events, defined as stroke, major bleeding, cardiovascular hospitalization, or cardiovascular death, as additional outcomes. Finally, we considered occurrence of any clinical event among those described previously as a study outcome. All the specific clinical events (ie, not those composite) were adjudicated centrally, according to the original study protocol.^20^

### Statistical Analysis

All continuous variables were reported as median and interquartile range (IQR). Categorical variables were expressed as counts and percentages and compared using the chi‐square test.

Cumulative incidence of adverse events is shown using Kaplan–Meier curves and compared across the groups with the log‐rank test. Cox regression was used to assess the association between the use of integrated care adherent to the ABC pathway and the occurrence of outcomes. Covariates considered for adjustment were age, sex, diabetes mellitus, hepatic/renal disease, pulmonary disease, first AF episode, and use of aspirin and were implemented in the various models as reported specifically in the tables and figures.

The main analyses included comparisons between the ABC pathway consistent group versus the non‐ABC pathway group. A secondary analysis examined the relationship between the total number of ABC pathway criteria fulfilled and occurrence of the study outcomes. Finally, a sensitivity analysis examined the degree of overlap between the 3 subgroups and the impact of the ABC adherent management. A 2‐sided *P*<0.05 was considered statistically significant. All analyses were performed using SPSS v. 25.0 (IBM, Armonk, NY).

## Results

From the original AFFIRM cohort, a total of 3169 (78.0%) patients were available for this analysis.^17^ Baseline characteristics of this cohort are reported in Table 1. Median (IQR) age was 70 (65–76) years, 1237 (39.0%) were female, median (IQR) CHA_2_DS_2_‐VASc score was 3 (2–4) and median (IQR) time in therapeutic range was 67.9% (51.5–81.0%). Of this cohort, 222 (7.0%) were managed consistent with the ABC pathway.^17^

**Table 1:** Baseline Characteristics of the Overall Cohort and Clinically Complex Subgroups

	Overall Cohort N=3169	Multimorbidity N=1723	Polypharmacy N=1222	Hospitalization N=1360
Age y, median (IQR)	70 (65–76)	70 (64–76)	71 (65–76)	70 (65–76)
Female sex, n (%)	1237 (39.0)	656 (38.1)	530 (43.4)	586 (43.1)
Hypertension, n (%)	2243 (70.8)	1445 (83.9)	1009 (82.6)	979 (72.0)
Diabetes mellitus, n (%)	625 (19.7)	576 (33.4)	325 (26.6)	306 (22.5)
Smoking, n (%)	378 (11.9)	256 (14.9)	167 (13.7)	179 (13.2)
Coronary artery disease, n (%)	1164 (36.7)	873 (50.7)	653 (53.4)	567 (41.7)
Myocardial infarction, n (%)	523 (16.5)	489 (28.4)	333 (27.3)	262 (19.3)
Peripheral arterial disease, n (%)	202 (6.4)	190 (11.0)	112 (9.2)	103 (7.6)
Stroke/TIA, n (%)	431 (13.6)	379 (22.0)	195 (16.0)	235 (17.3)
Heart failure, n (%)	684 (21.6)	659 (38.2)	442 (36.2)	393 (28.9)
Valvular heart disease, n (%)	401 (12.7)	354 (20.5)	192 (15.7)	177 (13.0)
Hepatic/renal disease, n (%)	158 (5.0)	149 (8.6)	88 (7.2)	87 (6.4)
Pulmonary disease, n (%)	427 (13.5)	375 (21.8)	199 (16.3)	226 (16.6)
First AF episode, n (%)	1016 (33.1)^a^	610 (36.5)^b^	419 (34.3)^c^	556 (43.0)^d^
Use of aspirin, n (%)	772 (24.4)	468 (27.2)	462 (37.8)	413 (30.4)
CHA_2_DS_2_‐VASc, median (IQR)	3 (2–4)	4 (3–4)	3 (2–4)	3 (2–4)
TTR %, median (IQR)	67.9 (51.5–81.0)	65.9 (48.1–80.0)	67.1 (49.3–80.8)	63.4 (46.3–79.2)
ABC pathway adherent patients, n (%)	222 (7.0)	66 (3.8)	49 (4.0)	65 (4.8)
Follow‐up time y, median (IQR)	3.70 (2.82–4.59)	3.63 (2.73–4.54)	3.59 (2.73–4.49)	3.78 (2.89–4.67)

ABC indicates Atrial fibrillation Better Care; AF, atrial fibrillation; IQR, interquartile range; TIA, transient ischemic attack; and TTR, time in therapeutic range.

a Available for 3067 patients.

b Available for 1673 patients.

c Available for 1222 patients.

d Available for 1292 patients.

The multimorbidity group comprised 1723 (54.4%) patients, 1222 (38.6%) were included in the polypharmacy group, and 1360 (42.9%) in the hospitalization group. Baseline characteristics for the 3 groups are summarized in Table 1. Median age was similar between the groups, with a slightly lower prevalence of females in the multimorbidity group. CHA_2_DS_2_‐VASc score was numerically higher in the multimorbidity group compared with the overall cohort and the other subgroups. ABC pathway consistent management was found in 66 (3.8%) in the multimorbidity group, 49 (4.0%) in the polypharmacy group, and in 65 (4.8%) for the hospitalization group.

### Follow‐Up Analysis

**Figure 1:**
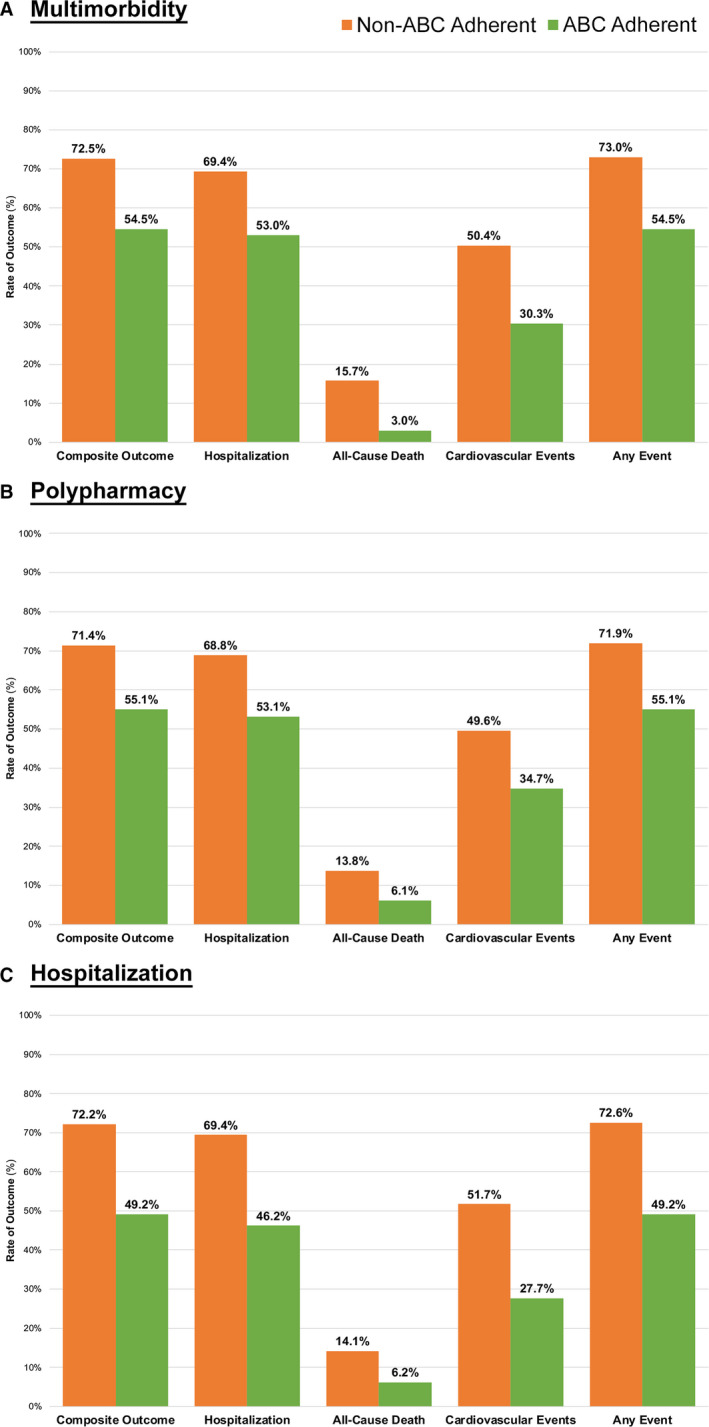
Rate of major adverse events according to clinically complex subgroups. **A**, Multimorbidity: composite outcome: *P*=0.001, hospitalization: *P*=0.005, all‐cause death: *P*=0.005, cardiovascular events: *P*=0.001, any event: *P*=0.001; (**B**) Polypharmacy: composite outcome: *P*=0.014, hospitalization: *P*=0.021, all‐cause death: *P*=0.123, cardiovascular events: *P*=0.041, any event: *P*=0.011; (**C**) Hospitalization: composite outcome: *P*<0.001, hospitalization: *P*<0.001, all‐cause death: *P*=0.068, cardiovascular events: *P*<0.001, any event: *P*<0.001. ABC indicates Atrial fibrillation Better Care.

In the multimorbidity group, after a median (IQR) 3.63 (2.73–4.54) years of follow‐up, there were 1238 composite outcome events (37.8 per 100 patient‐years), 1185 hospitalization events (36.2 per 100 patient‐years), 262 all‐cause death (4.21 per 100 patient‐years), 855 cardiovascular events (20.3 per 100 patient‐years), and a total of 1245 “any event” outcomes (38.3 per 100 patient‐years). Event rates for the non‐ABC group was significantly higher than the ABC group for all outcomes considered (Figure 1A).

In the polypharmacy group, after a median (IQR) 3.59 (2.73–4.49) years of follow‐up, a similar rate of events was evident, except for all‐cause death, which was numerically lower than in the multimorbidity group. There were 865 composite outcome events (37.7 per 100 patient‐years), 833 hospitalizations (36.3 per 100 patient‐years), 165 deaths (3.76 per 100 patient‐years), 599 cardiovascular events (20.5 per 100 patient‐years), and a total of 870 “any event” outcomes (38.4 per 100 patient‐years). The overall rate of outcomes was higher in non‐ABC pathway‐adherent group than in ABC pathway‐adherent group (see Figure 1), except for all‐cause death (*P*=0.123) (Figure 1B).

A similar rate of events was reported in the hospitalization group, with 967 composite outcome events (37.0 per 100 patient‐years), 929 recurrent hospitalization (35.5 per 100 patient‐years), 187 deaths (3.66 per 100 patient‐years), 687 cardiovascular events (20.7 per 100 patient‐years), and a total of 972 “any event” outcomes (37.5 per 100 patient‐years). Similar to the results from the other 2 groups, non‐ABC pathway adherent patients reported a higher rate of all outcomes under consideration (all *P*<0.001), except for all‐cause death; mortality was numerically lower but did not reach statistical significance (*P*=0.068) (Figure 1C).

**Figure 2:**
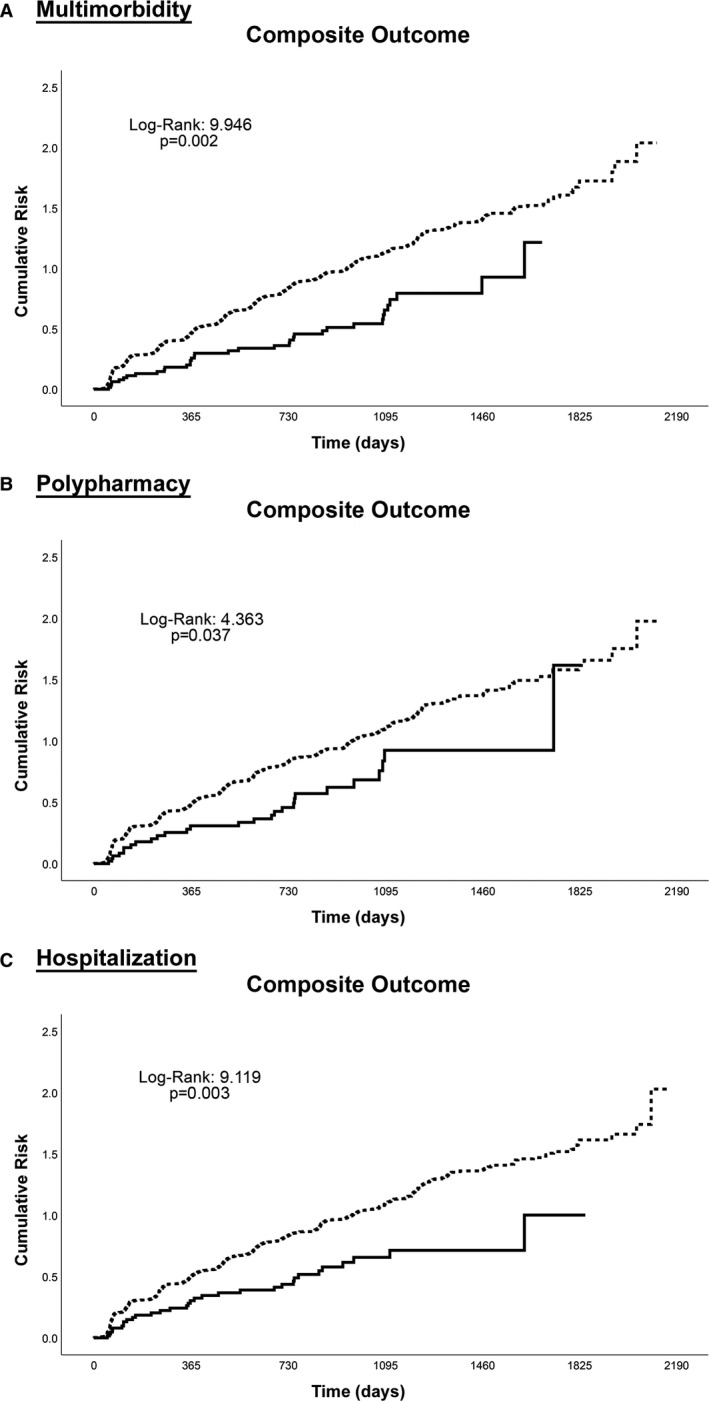
Kaplan–Meier curves for any event according to clinically complex subgroups. **A**, Multimorbidity; (**B**) Polypharmacy; (**C**) Hospitalization. ABC indicates Atrial fibrillation Better Care; dashed line, non‐ABC adherent; and solid line, ABC adherent.

Kaplan–Meier curves for the composite outcome showed that patients managed with ABC pathway‐adherent care had a lower cumulative risk in all 3 “clinically complex” patient groups (Figure 2A through 2C).

### Cox Regression Analysis

A Cox regression analysis was performed (Table 2). In the multimorbidity group, ABC clinical management was associated with a reduction in risk for the composite outcome (hazard ratio [HR], 0.61, 95% CI, 0.44–0.85, *P*=0.004), with a significant reduction in relative risk for all the other outcomes considered, in particular, for all‐cause death (HR, 0.23, 95% CI, 0.06–0.94, *P*=0.041) (Table 2).

**Table 2:** Relationship Between Integrated Care and Major Adverse Events

	ABC Adherent vs Non‐ABC Adherent			
	Univariate Analysis		Multivariate Analysis	
	HR (95% CI)	*P* Value	HR (95% CI)	*P* Value
Multimorbidity^a^				
Composite outcome	0.60 (0.43–0.83)	0.002	0.61 (0.44–0.85)	0.004
Hospitalization	0.61 (0.44–0.85)	0.004	0.62 (0.45–0.87)	0.006
All‐cause death	0.22 (0.06–0.88)	0.033	0.23 (0.06–0.94)	0.041
Cardiovascular events	0.53 (0.34–0.83)	0.005	0.54 (0.35–0.84)	0.007
Any event	0.59 (0.42–0.82)	0.002	0.60 (0.43–0.84)	0.003
Polypharmacy^b^				
Composite outcome	0.67 (0.46–0.98)	0.038	0.68 (0.47–1.00)	0.053
Hospitalization	0.68 (0.45–0.99)	0.042	0.69 (0.46–1.01)	0.058
All‐cause death	0.50 (0.16–1.56)	0.23	0.49 (0.16–1.54)	0.22
Cardiovascular events	0.66 (0.41–1.06)	0.087	0.67 (0.41–1.08)	0.099
Any event	0.66 (0.45–0.97)	0.033	0.68 (0.46–0.99)	0.045
Hospitalization^c^				
Composite outcome	0.59 (0.41–0.83)	0.003	0.59 (0.42–0.85)	0.004
Hospitalization	0.57 (0.40–0.82)	0.003	0.58 (0.40–0.84)	0.004
All‐cause death	0.51 (0.19–1.36)	0.18	0.49 (0.18–1.33)	0.16
Cardiovascular events	0.48 (0.30–0.76)	0.002	0.48 (0.30–0.77)	0.002
Any event	0.58 (0.41–0.83)	0.002	0.59 (0.41–0.84)	0.003

ABC indicates Atrial fibrillation Better Care; AF, atrial fibrillation; and HR, hazard ratio.

a Multivariate analysis adjusted for age, sex, first AF episode, use of aspirin.

b Multivariate analysis adjusted for age, sex, first AF episode, diabetes mellitus, hepatic/renal disease, pulmonary disease.

c Multivariate analysis adjusted for age, sex, first AF episode, diabetes mellitus, hepatic/renal disease, pulmonary disease, use of aspirin.

In the polypharmacy group, adjusted risk reduction for the composite outcome approached statistical significance (HR, 0.68, 95% CI, 0.47–1.00, *P*=0.053) (Table 2). A reduction in risk was found for any event outcome (HR, 0.68, 95% CI, 0.46–0.99, *P*=0.045), but no significant difference was found for all‐cause death (Table 2).

Results similar to those in the multimorbidity group were observed for patients hospitalized at the time of the index event, with a significant reduction in the risk of the composite outcome and other secondary outcomes, but no significant difference in the risk of all‐cause death was found (Table 2).

### Secondary Analysis

**Figure 3:**
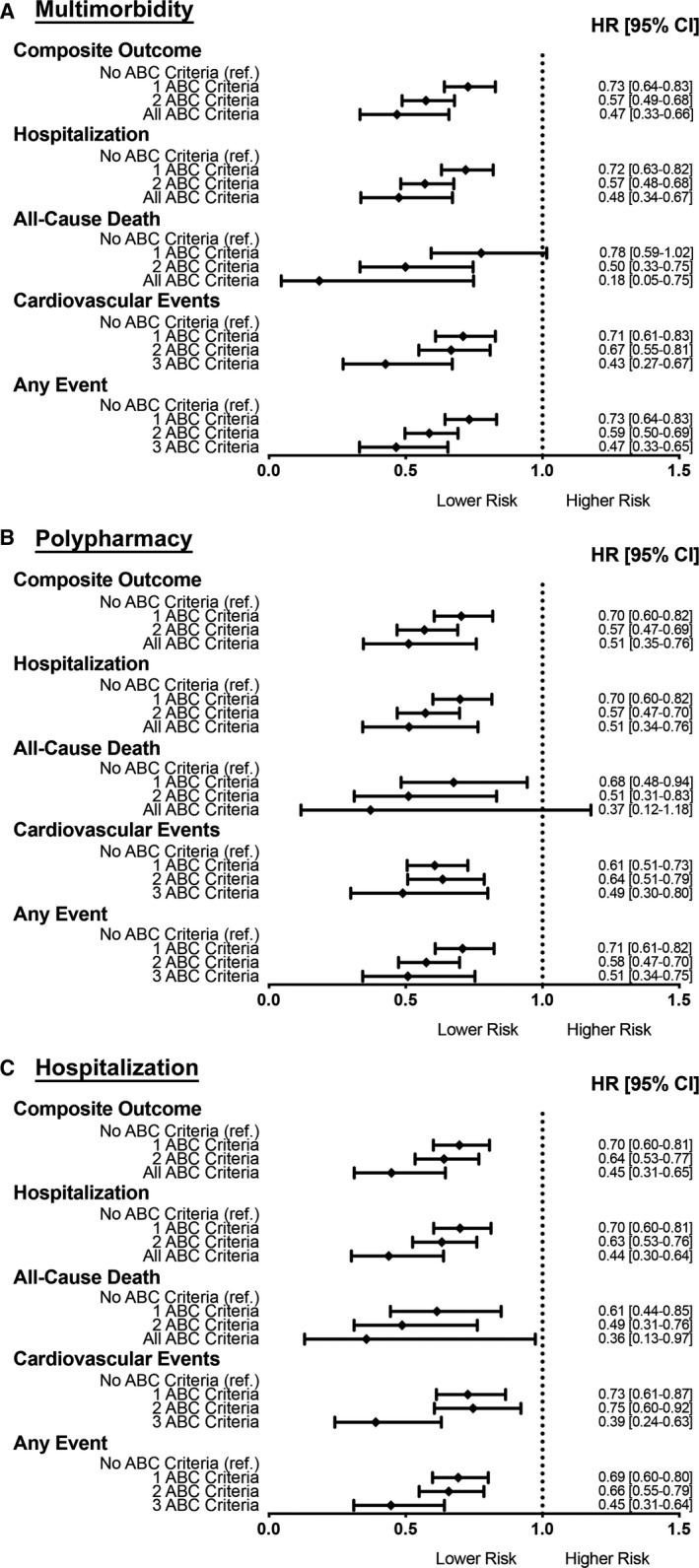
Relationship between number of ABC criteria and major adverse events according to clinically complex subgroups. **A**, Multimorbidity group analysis adjusted for age, sex, first AF episode, use of aspirin; (**B**) Polypharmacy group analysis adjusted for age, sex, first AF episode, diabetes mellitus, hepatic/renal disease, pulmonary disease; (**C**) Hospitalization group analysis adjusted for age, sex, first AF episode, diabetes mellitus, hepatic/renal disease, pulmonary disease, use of aspirin. ABC indicates Atrial fibrillation Better Care; and HR, hazard ratio.

A secondary analysis was performed to compare the number of ABC pathway criteria fulfilled versus no ABC pathway criteria fulfilled (Figure 3). In the multimorbidity group, there was a progressively lower risk of outcomes according to the increasing number of ABC criteria fulfilled (Figure 3A), with the higher risk reduction obtained when all 3 ABC criteria are fulfilled compared with no ABC criteria.

In the polypharmacy group a similar relationship between the number of ABC criteria and occurrence of outcomes was found, albeit with a slightly lower magnitude than in the multimorbidity group (Figure 3B). For the occurrence of the all‐cause death, a significant reduction in risk was associated with 1 ABC criterion (HR, 0.68, 95% CI, 0.48–0.94) or 2 ABC criteria fulfilled (HR, 0.51, 95% CI, 0.31–0.83); however, when all 3 ABC criteria were fulfilled there was a nonstatistically significant reduction, with wide 95% CI (HR, 0.36, 95% CI, 0.12–1.18) (Figure 3B).

In the hospitalization group, a similar reduction in the risk of major adverse events was evident with an increasing number of ABC criteria fulfilled, with the strongest reduction in risk for the composite outcome when all 3 ABC criteria were fulfilled (HR, 0.45, 95% CI, 0.31–0.65). In all 3 “clinically complex” patients’ subgroups, increasing number of ABC pathway criteria fulfilled were associated with a progressively lower risk for the occurrence of “any event,” with the exception of the outcome of all‐cause death in the polypharmacy group.

### Sensitivity Analysis

We examined how much the 3 subgroups overlapped and what was the impact of ABC adherent management in reducing the occurrence of outcomes. Among the 3169 patients included in this analysis, only 740 (23.4%) were not included in any of the 3 clinically complex subgroups, whereas among the remaining 2429 patients, 1058 (43.6%) were included in only 1 subgroup, 866 (35.7%) were included in 2 subgroups, and 493 (20.8%) were included in all 3 subgroups.

The prevalence of ABC pathway adherent group decreased progressively from those included in only 1 subgroup to those included in all 3 subgroups (7.4% versus 3.8% versus 2.4%, *P*<0.001). Given the low numbers, we considered the occurrence of only the primary outcome. Among those included in ≥2 subgroups, patients managed adherent to ABC pathway had a lower rate of composite outcome (57.8% versus non‐ABC adherent, 74.0%; *P*=0.015). After adjustment, the Cox regression analysis demonstrated that ABC pathway adherence among those included in ≥2 subgroups was associated to a lower risk of the primary outcome (HR, 0.60, 95% CI, 0.44–0.96).

## Discussion

In this post hoc subgroup analysis derived from the AFFIRM trial, we showed that in clinically complex patient subgroups (ie, multimorbidity, polypharmacy, hospitalization), management with an approach consistent with the one proposed by the ABC pathway, which streamlines the approach to AF patients’ care, was associated with reduction in the composite outcome of all‐cause hospitalization and all‐cause death in all the 3 groups considered. Management consistent with the ABC pathway was associated with reduction of “any event” outcome for those clinical events considered. Further, an increasing number of ABC criteria fulfilled was associated with a progressively larger reduction in risk for most of the outcomes considered. Lastly, even among patients having ≥2 clinical complex characteristics, the adherence to ABC pathway is still associated with a lower risk of the composite outcome, reinforcing the separate evidence coming from the 3 clinical subgroups.

The impact of the 3 clinical subgroups (ie, multimorbidity, polypharmacy, hospitalization) in determining an increased risk in major adverse events has been previously described.^6^, ^7^, ^8^, ^9^, ^10^, ^11^, ^12^, ^23^ For the presence of multimorbidity, several observational and randomized controlled trials have shown an increased risk for all major adverse events relevant to AF patients, in particular all‐cause death.^7^, ^8^, ^23^ Similarly, an increased risk of cardiovascular events and death is evident for AF patients reporting polypharmacy^6^, ^9^, ^10^ and an increased risk of death in hospitalized AF patients^11^, ^12^ were reported. In all these conditions, an increased rate of events was described, with an increased association with risk of events that was found to be independent of other clinical characteristics. Given the increased complexity and the higher risk of major adverse events in those with the described clinical features, an approach consistent with the ABC pathway may be beneficial. Indeed, a large group of patients were included in at least 2 of the subgroups and hence, had even greater clinical complexity.

Streamlining decision making to facilitate management of clinically complex patients with AF starting with primary care and linking with secondary care (including cardiologist and noncardiologists) may improve outcomes. The ABC pathway has been proposed as a simple and pragmatic approach to streamline and integrate care.^16^ Thus far, the ABC pathway has been tested indirectly, but it appears to reduce major adverse events in patients with AF.^17^, ^18^, ^19^ Prior studies have assessed several clinical settings and patients’ characteristics including the overall AFFIRM cohort.^17^, ^18^, ^19^

In our study, together with the previous analysis on the ABC pathway in AFFIRM,^17^ we found a low percentage of patients treated as fully adherent to the ABC pathway compared with other studies.^18^, ^19^ In a prospective Italian observational cohort, for example, management adherent with the ABC pathway was associated with a 56% risk reduction for a composite outcome of cardiovascular events.^18^ Similarly, in a population‐based nationwide cohort, there was a significant reduction (≈15%) in all adverse outcomes with ABC pathway‐adherent management.^19^ Considering the time of the AFFIRM study enrollment (>15 years ago), it is possible that a clinical management based on a single‐disease approach was more common in the past, compared with current practice, in which holistic and integrated management is more used.

In the present post hoc analysis, even in high‐risk subgroups, where the risk of adverse events is high, use of an ABC pathway approach was associated with fewer AF‐related outcomes. The adjudicated outcomes of hospitalization and all‐cause death were reduced in all 3 subgroups. The risk for hospitalization (considered as a single outcome) was also reduced with similar magnitude. Nonetheless, we may be underpowered to assess reduction in all‐cause death in the polypharmacy and hospitalization groups.

Our secondary analysis showed that risk was progressively lowered with a progressively higher numbers of ABC criteria fulfilled. This trend was also evident for all‐cause death in the polypharmacy and hospitalization groups. The low number of events in the fully ABC pathway‐adherent group is a limitation; however, the consistency of results in the patients with even higher clinical complexity reinforces the idea that the more complex patient is much more likely to get a beneficial effect from a comprehensive and integrated approach to AF care. Indeed, utilization of the ABC pathway was associated with reduction in “any clinical event” among these clinically complex patients, which further emphasizes the importance of improving the overall management of AF patients comprehensively, beyond thromboembolic risk.^24^

### Limitations

The post hoc nature, the modest number of subjects in the ABC compliant groups compared with the overall cohort (which limits the reliability of Kaplan–Meier analysis) and the relatively aged data set are limitations to this analysis. Because the AFFIRM study is an old clinical trial, this could limit the generalizability of our results, given the changes in AF management practices that have occurred in the past 15 years. Also, other general management aspects of AF patients (such as weight management, evaluation/management of sleep apnea, etc) were not routinely assessed at the time of the AFFIRM study and were not reported in the trial data set. The AFFIRM trial compared rhythm versus rate control, but this was not the objective of the present study, which focused on “better symptom” management overall (even within the heart failure subgroup, where rhythm control with catheter ablation has been beneficial compared with only drug therapy^25^). Nonetheless, we believe that the AFFIRM study design, which included patients with significant risk factors, still gives a good representation of the “typical” AF population.

Conversely, the adjudicated outcomes and the largely proved reliability of the AFFIRM database makes this *hypothesis generating* analysis reliable. Indeed, these results, supported by previous evidence, encourages use of an ABC pathway‐adherent approach, to streamline and integrate care in clinically complex patients with AF.

## Conclusions

Management of AF by an ABC consistent pathway is associated with reduction in major adverse events in clinically complex AF patients, including those with multimorbidity, polypharmacy, and prior hospitalization. These exploratory findings need further confirmation in larger, more contemporary studies.

## Sources of Funding

None.

## Disclosures

Proietti reports consulting activity for Boehringer Ingelheim; Olshansky has been consultant for Lundbeck, Amarin, Boehringer Ingelheim; Lane reports educational grants from Bristol‐Myers Squibb and Boehringer Ingelheim, speaker activity for Pfizer, and consultant activity for Bristol‐Myers Squibb, Bayer and Boehringer Ingelheim; Lip has served as consultant for Bayer/Janssen, BMS/Pfizer, Biotronik, Medtronic, Boehringer Ingelheim, Microlife, and Daiichi‐Sankyo. Speaker for Bayer, BMS/Pfizer, Medtronic, Boehringer Ingelheim, Microlife, Roche, and Daiichi‐Sankyo. No fees are received personally. The remaining authors have no disclosures to report.
